# Epigenetic Characterization of *CDKN1C* in Placenta Samples from Non-syndromic Intrauterine Growth Restriction

**DOI:** 10.3389/fgene.2016.00062

**Published:** 2016-04-26

**Authors:** Miriam López-Abad, Isabel Iglesias-Platas, David Monk

**Affiliations:** ^1^Servicio de Neonatología, Sant Joan de Déu, Centro de Medicina Maternofetal y Neonatal Barcelona, Hospital Sant Joan de Déu y Hospital Clínic, Universitat de BarcelonaBarcelona, Spain; ^2^Imprinting and Cancer group, Cancer Epigenetic and Biology Program, Institut d’Investigació Biomedica de BellvitgeBarcelona, Spain

**Keywords:** imprinting, DNA methylation, placenta, *CDKN1C*, epigenetics

## Abstract

The cyclin-dependent kinase (CDK)-inhibitor 1C (*CDKN1C*) gene is expressed from the maternal allele and is located within the centromeric imprinted domain at chromosome 11p15. It is a negative regulator of proliferation, with loss-of-function mutations associated with the overgrowth disorder Beckwith–Wiedemann syndrome. Recently, gain-of-function mutations within the PCNA domain have been described in two disorders characterized by growth failure, namely IMAGe (intra-uterine growth restriction, metaphyseal dysplasia, adrenal hypoplasia congenita and genital abnormalities) syndrome and Silver–Russell syndrome (SRS). Over-expression of *CDKN1C* by maternally inherited microduplications also results in SRS, suggesting that in addition to activating mutations this gene may regulate growth by changes in dosage. To determine if *CDKN1C* is involved in non-syndromic IUGR we compared the expression and DNA methylation levels in a large cohort of placental biopsies from IUGR and uneventful pregnancies. We observe higher levels of expression of *CDKN1C* in IUGR placentas compared to those of controls. All placenta biopsies heterozygous for the PAPA repeat sequence in exon 2 showed appropriate monoallelic expression and no mutations in the PCNA domain were observed. The expression profile was independent of both genetic or methylation variation in the minimal *CDKN1C* promoter interval and of methylation of the *cis*-acting maternally methylated region associated with the neighboring *KCNQ1OT1* non-coding RNA. Chromatin immunoprecipitation revealed binding sites for CTCF within the unmethylated *CDKN1C* gene body CpG island and putative enhancer regions, associated with the canonical enhancer histone signature, H3K4me1 and H3K27ac, located ∼58 and 360 kb away. Using 3C-PCR we identify constitutive higher-order chromatin loops that occur between one of these putative enhancer regions and *CDKN1C* in human placenta tissues, which we propose facilitates expression.

## Introduction

Intrauterine growth restriction (IUGR) is a condition in which a fetus is unable to achieve its genetically determined *in utero* size and is associated with increased risk of perinatal morbidity and mortality. The mechanisms that lead to IUGR are not completely understood. Etiologically, restricted growth can be of fetal, maternal and placental origin. Aberrant maternal-fetal circulation has been consistently implicated because of the observed abnormalities in Doppler dynamics of the uterine and umbilical arteries, which indicate increased resistance in the maternal spiral arteries and the placental circulation, respectively (Society for Maternal-Fetal Medicine Publications Committee et al., 2012). Pregnancies that are complicated by IUGR often require early elective delivery due to higher risk of fetal distress, resulting in additional complications associated with prematurity (Miller et al., 2009) which require prolonged admission in neonatal intensive care.

Chromosomal abnormalities are often a cause of severe growth restriction (Romero et al., 2015). With the advent of genome-wide technologies, altered gene expression profiles in the fetus or placenta are commonly being described in IUGR and other pregnancy complications. These abnormal expression profiles are often associated with increased epigenetic variance, suggesting links between underlying chromatin dynamics and fetal growth. In particular, imprinted genes, of which there are ∼150 described in the human genome, have been shown to be essential for appropriate fetal and placenta development. Emerging evidence implicates aberrant expression levels of imprinted genes in not only classical imprinting disorders (reviewed in Eggermann et al., 2015), but also in many common multifactorial human diseases, which include complications of pregnancy such as IUGR, pre-eclampsia (reviewed in Monk, 2015) and postnatal disorders including obesity and type 2 diabetes (Kong et al., 2009).

### Genomic Imprinting

Imprinted genes encode products implicated in diverse physiological processes, many of them playing a role in growth and development. The molecular mechanisms regulating genomic imprinting involve the establishment of parent-specific epigenetic modifications in the germline, which result in monoallelic expression of transcripts in a parent-of-origin dependent manner (Ferguson-Smith, 2011). Until recently, it was thought that imprinted genes have a tendency to cluster together as a result of sharing *cis*-regulatory elements, including differentially methylated regions (DMRs) that inherit methylation from one of the two gametes. However, recent genome-wide screens for novel imprinted loci have identified placenta-specific maternally methylated DMRs that are more prevalent in the human genome than ubiquitous imprinted domains (Court et al., 2014b), and that do not orchestrate imprinting of neighboring genes (Sanchez-Delgado et al., 2015).

#### The *KCNQ1OT1* Imprinted Domain

**FIGURE 1:**
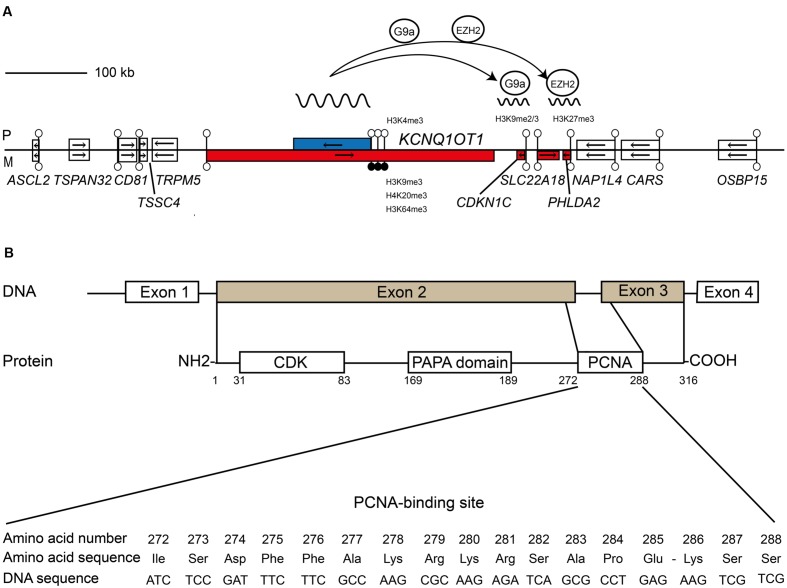
**Schematic representation shows the relative organization of genes, CpG islands and differentially methylated regions (DMRs) for *KCNQ1OT1*-*CDKN1C* domain. (A)** The centromeric imprinting domain on human chromosome 11p15.5 highlighting the role of the ncRNA *KCNQ1OT1* in recruiting the G9a and EZH2 histone methyltransferase complexes to the paternal allele of *CDKN1C*. Open boxes depict non-imprinted genes. Blue boxes are paternally expressed genes; red boxes are maternally expressed genes, respectively. Lollipops signify CpG islands with open circles unmethylated and black circles methylated. The arrows show the direction of transcription. **(B)** Structural features and functional domains of the *CDKN1C* gene and protein. The *CDKN1C* possesses four exons with exons 2 and 3 encoding the functional protein. The protein has three key domains: a conserved amino-terminal containing the CDK inhibitory domains; the proline–alanine repeat (PAPA) domain; and the proliferating cell nuclear antigen (PCNA) domain which contains a nuclear localization signal. The nucleotide and amino acid sequence of the PCNA domain is shown.

Distal mouse chromosome 7 harbors the largest known imprinted cluster and is highly conserved in humans. Two germline DMRs control different sets of imprinted transcripts within this >1 Mb cluster, divided into two functionally independent domains. The centromeric domain is controlled by the paternally methylated *H19/IGF2:*IG-DMR (note adoption of recommended DMR name as recommended by the European Network for Human Congenital Imprinting Disorders – EUCID.net^1^. Also known as the *H19* ICR or ICR1; Thorvaldsen et al., 1998), whereas the telomeric domain is regulated by the *KCNQ1OT1:*TSS-DMR (also known as *Kv*DMR1 or ICR2; Lee et al., 1997; Caspary et al., 1998). This second DMR is a CpG island within intron 10 of the *Kcnq1/KCNQ1* gene that inherits methylation from oocytes (Hiura et al., 2006) and is the promoter for the long ncRNA *KCNQ1OT1* (also known as *LIT1*). In the mouse, this long ncRNA recruits the histone methyltransferases G9a and EZH2 to the promoters of the flanking genes depositing trimethylation of lysine 27 of histone H3 (H3K27me3) and H3K9me2 (Lewis et al., 2004; Umlauf et al., 2004; Mohammad et al., 2012) on the paternal allele bring about imprinting in the placenta (Okae et al., 2012). Truncation of *Kcnq1ot1* results in loss of imprinted expression of all genes in the placenta, confirming that the products of transcription are essential for imprinting maintenance (Mancini-Dinardo et al., 2006). Interestingly, despite conserved expression of *KCNQ1OT1*, imprinting of the placenta-specific transcripts within the human orthologous domain is not conserved, since their promoters are not decorated with allelic repressive histone modifications (Monk et al., 2006) (**Figure 1A**). However three genes, *CDKN1C*, *PHLDA2*, and *SLC22A18* are imprinted, being monoallelically expressed in both placenta and fetal tissues (Yatsuki et al., 2002; Monk et al., 2006).

### The *CDKN1C* Gene

Cyclin-dependent kinase (CDK) Inhibitor 1C (also known as *p57Kip2*) has four exons and was first discovered as a result of sequence similarity with other CDK inhibitors in a two-hybrid screening (Hatada and Mukai, 1995; Matsuoka et al., 1995). *CDKN1C* mRNA transcripts are found in placenta, heart, brain, lung, skeletal muscle, kidney, pancreas, and testis by northern analysis (Matsuoka et al., 1995). Microarray expression analysis reveals that *CDKN1C* is most abundant in placenta compared to somatic tissues (Sood et al., 2006). The protein has highly conserved N-terminal CDK inhibition and C-terminal domains. The human protein, but not the mouse, also contains a proliferating cell nuclear antigen (PCNA) binding domain required for preventing DNA replication (Watanabe et al., 1998). In addition *CDKN1C* has a QT-box involved in protein-protein interactions and a unique proline-rich and acidic domain in the mouse, replaced with proline–alanine (PAPA) repeats in humans, which are involved in mitogen-activated protein kinase (MAPK) phosphorylation (Lee et al., 1995). The absence of conserved sequence in the mid-region of the protein could be attributable to the lack of function, merely acting as a spacer region to separate the functional N- and C-terminal domains (Matsuoka et al., 1995; Tokino et al., 1996) (**Figure 1B**).

#### *CDKN1C* Cell Cycle Function

CDKN1C is one of the CDKs inhibitors belonging to the Cip/Kip family, which includes CDKN1A (p21cip1) and CDKN1B (p27kip1). These proteins are required for cell cycle transition and play important roles in coordinating cell proliferation, differentiation and maintenance of the non-proliferative state of cells. *Cdkn1c* is primarily expressed in cells that are exiting cell cycle but are not terminally differentiated and shows specificity for G1 CDKs. It can bind several CDKs in a cyclin-dependent manner, including cyclin A/Cdk2, cyclin E/Cdk2, cyclin E/Cdk3, cyclin D2/Cdk4 and, to a lesser extent, cyclin D2/Cdk6. Furthermore, CDKN1C can inhibit the kinase activity of the G1 cyclin Cdk2, Cdk3, and Cdk4 complexes. Therefore CDKN1C is capable of inhibiting several CDK with demonstrated roles in the G1/S-phase transition. However, in mouse embryonic fibroblasts and placenta from null animals, disruption of the protein does not affect the activities of Cdk2 and Cdk4 (Takahashi et al., 2000), suggesting that Cdkn1c may have a biological activity other than inhibition of Cdk activities, presumably linked with PCNA-associated DNA replication regulation (Watanabe et al., 1998), thus affecting cell cycle regulation via different mechanisms.

#### Mutations of Human *CDKN1C* and Growth

In addition to large copy-number alterations of 11p15 that lead to developmental phenotypes (Begemann et al., 2012; Cerrato et al., 2014), mutations of *CDKN1C* also give rise to growth abnormalities. Since *CDKN1C* functions as a negative regulator of cellular proliferation, aberrations in this gene are predicted to result in over-proliferation and predisposition to cancer. Indeed, maternally inherited loss-of-function mutations are associated with 5% of sporadic and 50% of familial cases of Beckwith–Wiedemann syndrome (BWS; Lim and Maher, 2010; Eggermann et al., 2014). These genetic alterations in BWS are rare compared to epimutations of the *KCNQ1OT1*:TSS-DMR that results in overexpression of the *KCNQ1OT1* ncRNA and concomitant repression of both alleles of *CDKN1C* (Diaz-Meyer et al., 2003).

Recent reports of maternally inherited gain-of-function point mutations within the PCNA-binding domain are associated with the growth restriction disorders IMAGe (Arboleda et al., 2012) and Silver–Russell syndromes (SRS; Brioude et al., 2013). This domain is required for PCNA-dependent and CRL4Cdt2-mediated ubiquitination of three lysine residues, Lys278, Lys280, and Lys286 (Kirchmaier, 2011), with mutations presumably increasing the stability of the protein (Hamajima et al., 2013). Interestingly, expression of IMAGe-associated *CDKN1C* mutations does not interfere with the ability of CDKN1C to inhibit the cell cycle in phase G0/G1 through binding of the CDK, suggesting that domain-specific mutations have differential effects on cell-cycle progression and developmental processes (Arboleda et al., 2012).

#### *CDKN1C* and Placenta Development

During mid-to-late mouse placental development *Cdkn1c* is expressed in giant trophoblast cells, glycogen cells within the junctional zone and the syncytiotrophoblast (Westbury et al., 2001; Tunster et al., 2011). Mice that express *Cdkn1c* at twofold the normal endogenous level are growth restricted from embryonic day E13.5, whereas mice deficient for *Cdkn1c* were heavier at the same time point (Andrews et al., 2007). Studying growth dynamics reveals that *Cdkn1c* mutant embryos are ∼15% heavier than wild-type embryos at E15.5, 8% heavier at E18.5, with the difference diminishing until it is no longer apparent at birth (Tunster et al., 2011). Placentae of *Cdkn1c* homozygotes and heterozygous mice inheriting a targeted deletion from their mothers are larger than those of wild-type mice. This enlargement was associated with prominent proliferation of the labyrinthine and spongiotrophoblasts layers, disordered vascularization and glycogen storage (Takahashi et al., 2000) with substantial thrombotic lesions (Tunster et al., 2011). The severe placental abnormalities observed in *Cdkn1c* mutant mice preceded the attenuated overgrowth described previously. This loss of growth potential late in gestation is a classic indicator of placental insufficiency. Furthermore, the overgrowth of mutant mice decreased in the face of increasing intrauterine competition, suggesting that *Cdkn1c* is involved in the allocation of the maternal resources via the placenta (Tunster et al., 2011).

It is currently unknown if such dosage effects are observed in humans, since the human placenta has a hemochorial structure lacking a cell type equivalent to the giant trophoblast cell in mice (Carter and Enders, 2004). Therefore the role of *CDKN1C* in the human placenta might differ from that in the mouse. Several small studies have investigated changes in *CDKN1C* expression in human placenta cohorts with heterogeneous clinical characteristics (healthy pregnancies, preeclampsia, small for gestational age, and IUGR), with consistent up-regulation observed in IUGR suggesting that deregulation of this genes might play a role in prenatal growth (McMinn et al., 2006; Cordeiro et al., 2014). To further clarify the role of *CDKN1C* in fetal growth we have performed placental expression and DNA methylation profiling in normal and non-syndromic IUGR placental tissues. Combined interrogation of publicly available CTCF ChIP-seq and ChIA-PET data with molecular confirmation in placenta shows that *CDKN1C* physically interacts with putative long-range enhancer regions that we propose form constitutive chromatin loops and regulate expression.

## Materials and Methods

### The Placenta Cohort

Seventy-seven pregnant women delivering their babies in Sant Joan de Déu Hospital, Barcelona, participated in the study, contributing 89 newborns. The protocol was approved by both the Sant Joan de Déu Hospital and IDIBELL Research and Ethics Committees (PI35/07 and PR006/08) and individual informed consent was obtained. Upon delivery, the placentae were weighed and biopsies from the fetal side adjacent to the umbilical cord insertion site were excised. The tissue was thoroughly rinsed in saline and snap frozen in liquid nitrogen. Maternal blood samples were collected in EDTA tubes and frozen at –20°C until processed. Clinical information on pregnancy course for the anonymized samples was recorded. For the purpose of the analysis, subgroups were established according to the characteristics of the pregnancy and of the newborn (term or preterm, IUGR or non-IUGR, conceived spontaneously or by assisted reproduction). According to length of gestation, newborns were classified in: term (≥37 weeks), late and moderate preterm (>32 and <37 weeks) and very preterm (≤32 weeks). IUGR was defined as a weight below the third percentile for gestational age or below the 10th percentile when accompanied by fetal Doppler flow abnormalities (**Tables 1–3**).

**Table 1:** Clinical characteristics of the newborns in the sample.

Newborn characteristics	*n* (%)		*p*-value
	IUGR (*n* = 36)	Control (*n* = 53)	
Gender: boys	14 (38.9)	28 (52.8)	0.196
Gestational age group (w = weeks)			
Term (≥37 w)	13 (36.1)	21 (39.6)	
Late preterm (34 + 1 – 36 + 6 w)	11 (30.6)	7 (13.2)	0.226
Moderate premature (32 + 1 – 34 w)	6 (16.7)	11 (20.8)	
Very premature (≤32 w)	6 (16.7)	14 (26.4)	
Multiple gestation	11 (30.6)	19 (35.8)	0.604
No previous gestation	21 (58.3)	36 (67.9)	0.355
Conception by ART	11 (30.6)	17 (32.1)	0.880
Preeclampsia	8 (22.2)	2 (3.8)	0.014^∗^
Labor	18 (50.0)	32 (60.4)	0.333
Delivery by cesarean section	23 (63.9)	30 (56.6)	0.492
	**Mean ± SD**		
Gestational age (w)	35.1 ± 3.5	33.8 ± 7.3	0.274
Anthropometric data at birth			
Birth weight (g)	1841 ± 564	2340 ± 952	<0.003^∗^
Birth weight SDS	-1.55 ± 0.68	0.22 ± 0.72	<0.0001^∗^
Length (cm)	43.0 ± 4.3	45.0 ± 6.2	0.123
Length SDS	-1.26 ± 1.07	0.21 ± 0.68	<0.0001^∗^
Head circumference (HC, cm)	30.4 ± 2.6	31.5 ± 4.3	0.190
HC SDS	-1.22 ± 0.82	0.20 ± 0.76	<0.0001^∗^
Placental weight (g)	396 ± 133	578 ± 203	<0.0001^∗^
Birth weight to placenta ratio	4.89 ± 1.20	4.25 ± 1.29	0.034^∗^

*Standard deviation score (SDS), a variable that represent the distance of a data point to the mean of the distribution as measured in SDs. Values are expressed as number (n) and percentage and were compared between groups by Chi-square tests. Continuous variables are summarized as mean ± standard deviation and Student’s t-testing was used for statistical comparison. ^∗^P < 0.05 were considered significant. Note, since there are methylation differences in the placenta that are due to gestational age both IUGR and control patients were recruited amongst term and preterm deliveries to minimize the difference between groups regarding this parameter.*

**Table 2:** Maternal data.

Maternal characteristics	*n* (%)
Ethnicity: Caucasian	55 (71.4)
Parity: primiparous	49 (63.6)
Assisted reproduction	19 (24.7)
Previous obstetric history	
None	53 (68.8)
Infertility	12 (15.6)
Gestational hypertension/Pre-eclampsia	3 (3.9)
Recurrent miscarriage (≥3)	3 (3.9)
Previous IUGR	1 (1.3)
Other	2 (2.6)
Missing	3 (3.9)
Multiple gestation	19 (24.7)
Cesarean section	41 (53)
Pre-eclampsia	9 (11.7)
Pre-existing conditions	
None	58 (75.3)
Chronic hypertension	5 (6.5)
Psychologic/psychiatric disorders	2 (2.6)
Gestational age group	
Term (≥37 w)	34 (44.2)
Late preterm (34 + 1 – 36 + 6 w)	13 (16.9)
Moderate preterm (32 – 34 w)	14 (18.2)
Very preterm (≤32 w)	16 (20.8)
	**Mean ± SD**
Age at delivery (years)	32.5 ± 6.0
Height (cm)	162.6 ± 7.1
Weight (kg)	
Pre-pregnancy	64.9 ± 15.4
At delivery	76.7 ± 14.8
Gestational age at delivery (weeks)	35.0 ± 4.7

*Quantitative values are expressed as number (n) and percentage. Continuous variables are summarized as mean ± standard deviation.*

**Table 3:** Maternal data by study group.

Maternal characteristics	*n* (%)		*p*-value
	IUGR (*n* = 34)	Control (*n* = 43)	
Ethnicity: Caucasian	27 (79.4)	28 (65.1)	0.489
Parity: primiparous	20 (58.8)	29 (67.4)	0.435
Assisted reproduction	9 (26.5)	10 (23.3)	0.794
Previous obstetric history	11 (33.3)	10 (24.4)	0.396
Singleton gestation	9 (26.5)	10 (23.3)	0.745
Cesarean section	21 (61.8)	20 (46.5)	0.183
Labor	18 (52.9)	31 (27.9)	0.083
Pre-eclampsia	8 (23.5)	1 (2.3)	0.009^∗^
Healthy before pregnancy	22 (68.8)	36 (87.8)	0.046^∗^
Chronic hypertension	4 (12.5)	1 (2.4)	0.161
Gestational age group			
Term (≥37 w)	13 (38.2)	21 (48.8)	
Late preterm (34 + 1 – 36 + 6w)	11 (32.4)	2 (4.7)	
Moderate preterm (32 – 34 w)	5 (14.7)	9 (20.9)	
Very preterm (≤32 w)	5 (14.7)	11 (25.6)	
Age at delivery (years)	33.2 ± 6.3	32.0 ± 5.8	0.428
Height (cm)	162.1 ± 7.0	163.0 ± 7.3	0.621
Weight (kg)			
Pre-pregnancy	63.0 ± 15.7	66.3 ± 15.2	0.390
At delivery	73.9 ± 15.2	79.0 ± 14.3	0.181
Gestational age at delivery (weeks)	35.5 ± 3.6	34.6 ± 5.5	0.401

*Mothers were classified in the IUGR group if any of the babies in the current gestation fulfilled diagnostic criteria for IUGR. Quantitative values are expressed as number (n) and percentage and were compared between groups by Chi-square tests. Continuous variables are summarized as mean ± standard deviation and Student’s t-testing was used for statistical comparison. ^∗^P < 0.05 were considered significant.*

#### Other Tissues and Cell Lines

Control lymphoblastoid cell lines were established by EBV transformation of peripheral blood cells and propagated in RPMI media supplemented with 10% fetal calf serum (FCS) and antibiotics. The human TCL1 placental trophoblast cell line was grown in Dulbecco’s modified Eagle’s medium supplemented with 10% FCS and antibiotics. Prior to ChIP and 3C, the cell line 11p15 methylation signatures were compared to normal leukocytes and placenta samples to ensure that the transformation process had not altered the epigenetic profile.

### Nucleic Acid Extraction

Genomic DNA from placenta samples was prepared by SDS/Proteinase K lysis followed by phenol/chloroform extraction and ethanol precipitation. DNA from blood and cell lines was extracted using a commercial kit (QIAamp DNA Blood Midi Kit^®^, QIAGEN), following the manufacturer’s instructions. Total RNA was extracted using Trizol^®^ (Invitrogen), and 1 μg of RNA was treated with DNase (amplification grade DNase I, Invitrogen) prior to RT. Reverse transcription was performed with MMLV retrotranscriptase (Promega) and random primers (Promega) following the manufacturer’s instructions.

### Genotyping PCR Reactions

Sequences were obtained for all DNA samples using PCR and direct sequencing of the resulting amplicons using primers for the PCNA domain of *CDKN1C*, the minimal promoter of *CDKN1C* and the putative enhancer regions (see **Supplementary Table S1** for primer sequences). Approximately 200 ng genomic DNA was amplified using BioTaq DNA Polymerase (Bioline). PCR was performed for 35 cycles of denaturation at 94°C for 30 s, followed by 30 s annealing and extension at 72°C for 60 s. PCR amplicons were purified using ethanol precipitation and subsequently sequenced with the BigDye Terminator reaction kits on an ABI 3730 DNA analyser (PE Biosystems).

#### Quantitative Polymerase Chain Reaction

Expression of *CDKN1C* was analyzed by quantitative real-time RT-PCR with a fluorochrome (SYBR^®^ Green) assay and normalized against *RPL19* (Iglesias-Platas et al., 2014). This housekeeping gene was selected because of optimal expression stability in placental tissue compared to other commonly used housekeeping genes (**Supplementary Table S2**). Primers were designed in different exons or across intron/exon boundaries to avoid amplifying contaminating genomic DNA (see **Supplementary Table S1** for primer sequences). The assays were run in triplicate in 384 well plates in a 7900HT Fast Real Time PCR System (Applied Biosystems). Dissociation curves were obtained at the end of each reaction to rule out the presence of primer dimers or unexpected DNA species in the reaction. Non-template controls, an interplate control and standard curves from the same serial dilutions of cDNA obtained from pooled normal placental tissue were included in each assay. Results were analyzed with the SDS 2.3 software (Applied Biosystems). The DataAssist v2.0^®^ software (Applied Biosystems) was used for exclusion of outlier replicates and for interplate standardization for comparisons. Only samples with two or more valid readings per triplicate were included. Analysis of the results was performed using the comparative ΔΔCT (RQ) method (Schmitthen and Livak, 2008).

#### Assessment of Imprinted Expression

Analysis of allelic expression was determined using PCR across the polymorphic PAPA repeat in the second exon of *CDKN1C* (see **Supplementary Table S1** for primer sequences). The PCRs were performed with Immolase Taq polymerase (Bioline) for 40 cycles with an annealing temperature of 60°C. The resulting PCR products were separated on a 4% agarose gel stained with SYBR safe (Thermo Fisher Scientific). All placental DNA samples were genotyped to identify heterozygous individuals. Expression was then analyzed in heterozygous samples by RT-PCR with imprinting confirmed if a single band was observed in the RT-PCR product of a heterozygous sample. In these samples, the parental origin of expression was determined, when possible, by assessing the maternal genotype. In addition, RT-PCR was performed on RT-positive and negative samples in order to rule out genomic contamination.

#### Allele-Specific Bisulfite PCR

Approximately 1 μg DNA was subjected to sodium bisulfite treatment and purified using the EZ GOLD methylation kit (ZYMO, Orange, CA, USA) and ∼50 ng of converted DNA used for all bisulfite PCR reactions. Bisulfite PCR primers for each region were used with Immolase Taq polymerase (Bioline, London, UK) at 45 cycles with an annealing temperature of 53°C (see **Supplementary Table S1** for primer sequences). The resulting PCR product was cloned into pGEM-T easy vector (Promega), transfected into JM109 bacteria and individual colonies sequencing using standard T7 sequence primer.

#### Methylation Analysis by Bisulfite Pyrosequencing

Bisulfite treatment of 1 μg of DNA was performed with the EZ Gold in a 96-well plate format (EZ-96 DNA Methylation-GoldTM^^®^^ Kit, Zymo Research), following the manufacturer’s protocol. A commercial control was used as reference for bisulfite-converted fully methylated DNA (EpiTect Control DNA^®^, methylated, Qiagen). Pyrosequencing was selected for the quantitative assessment of DNA methylation at the *KCNQ1OT1:*TSS-DMR, the *CDKN1C* promoter and enhancer regions using standard primers with the exception that the reverse primer was biotin-labeled. Immobilization of the PCR products for purification was achieved by streptavidin-coated sepharose beads (Qiagen) with the use of the PyroMark Q96 Vacuum Prep Workstation^®^ and sequenced using a PyroMark Q96 MD machine according to the manufacturer’s instructions.

### Western Blot Analysis

Proteins from placenta samples with opposing *CDKN1C* transcript abundance as determined by qRT-PCR were extracted in standard lysis buffer (50 mM Tris 7.5 pH, 150 mM NaCl, 1 mM EDTA) containing 1% SDS with sonication to disrupt genomic DNA. Twenty-five micrograms β-mercaptoethanol denatured lysates were separated by PAGE gel electrophoresis and blotted onto a nitrocellulose transfer membrane (Whatman, Life Sciences). The membrane was blocked in 5% milk PBS-T and immunoprobed with antibodies raised against CDKN1C (Abcam anti-p57kip2: ab75974) and β-actin (Sigma: A1978). Washed membranes were incubated with corresponding peroxidase-conjugated secondary antibody. The immunoreactive proteins were visualized using the Immobilon chemiluminescent HRP substrate detection kit (Millipore). Bands were quantitated by direct scanning of the western blot films and processed with ImageJ software.

### Chromatin Immunoprecipitation

One hundred micrograms of snap frozen placental tissue were reduced to powder with a pestle and mortar under liquid nitrogen. The pulverized placenta sample was cross-linked with 1% formaldehyde for 7 min at room temperature and the reaction blocked by adding glycine to a final concentration of 0.125 M. Similar cross-linking protocols were used for ∼80 million lymphoblastoid cells. Approximately 100 μg chromatin were used for each immunoprecipitation reaction with Protein G magnetic beads (Millipore, 16–157) and an antibody raised against CTCF (Millipore, 07–729). For each ChIP, a fraction of the input chromatin (5%) was also processed for DNA purification and a mock immunoprecipitation with a neutral, unrelated IgG (Millipore PP64B Lot: 1968270) antiserum was carried out in parallel. The levels of immunoprecipitated chromatin at specific regions were determined by standard PCR (see **Supplementary Table S1** for primer sequences).

### 3C Analysis

The chromatin conformation capture (3C) protocol was performed as previously described (Court et al., 2014a; Iglesias-Platas et al., 2014). Briefly, HindIII was used to digest 1 × 10^7^ formaldehyde cross-linked nuclei from the placental cell line TCL1 (overnight digestion, 1200 U, NEB). Following efficiency restricted enzyme digestion the chromatin was ligated overnight in a 500 μl reaction volume using 1950 units of T4 ligase (Fermentas). DNA was decross-linked by incubating overnight at 65°C and purified using phenol/chloroform extraction. This DNA was used for PCR to determine long-range chromatin interactions, using constant primers in the unmethylated *CDKN1C* gene body CpG island (see **Supplementary Table S1** for primer sequences). Primer efficiency was determined using digested and ligated BAC DNA. All 3C experiments were performed in two technical replicates.

### Statistical Analysis

Clinical and molecular data were introduced in a Statistical Package for Social Sciences (SPSS^®^, IBM) software v17.0 database. Expression levels were expressed in logarithmic scale in order to achieve a variable distribution closer to normality and subsequently analyzed against clinical values. Continuous variable are summarized throughout the manuscript as mean ± standard deviation (SD) and qualitative variables as number and percentage. Comparisons between groups were evaluated with chi-square for categorical variables and *t*-Student test (for two groups, 2-tailed analysis) or ANOVA (more than two groups) for continuous variables. Non-parametric tests were applied where indicated. Relationships between variables were explored by Pearson’s correlation and subsequently introduced in multiple regression models to adjust for possible interactions or confounding factors. Results were considered significant if the *p*-value was under 0.05. This study was sufficiently powered to detect differences in expression of 0.7 SDs between groups (statistical power 80%) and correlations with a Pearson’s r coefficient greater than 0.3 between gene expression and clinical variables (statistical power 86%).

## Results

### Cohort Description

Eighty-nine placental samples were obtained from 77 pregnancies. The samples corresponded to 15 twin pregnancies (both newborns analyzed in eight sets and only one in the other seven) and two triplet pregnancies contributing six newborns to the study.

Most mothers (71.4%) were of Caucasian origin and were healthy before pregnancy. Characteristics of the case and the control groups are summarized in **Tables 1–3**. Due to the high-risk obstetric characteristics of our population (IUGR, prematurity, complications of pregnancy), there was a high rate of cesarean section (53%).

Thirty-six of the fetuses were diagnosed with IUGR during pregnancy. Mothers of IUGR babies had a higher prevalence of preeclampsia (23.5% vs. 2.3%, Chi-square *p* = 0.01) or chronic hypertension. There were no differences regarding primiparity (58.3% vs. 67.9%, Chi-square *p* = 0.35), delivery by cesarean section (63.9% vs. 56.6%, Chi-square *p* = 0.49) or in the percentage of mothers undergoing labor (50.0% vs. 60.4%, Chi-square *p* = 0.33) between IUGR and normally grown gestations. Conception by ART was equally frequent in both groups (30.6% vs. 32.1%, Chi square *p* = 0.88). IUGR and normally grown fetuses were comparable in their remaining clinical characteristics except, by definition, size for gestational age at birth (Student’s *t p* < 0.001) and the incidence of detection of Doppler flow abnormalities (Chi-square *p* < 0.001).

### Increased Levels of *CDKN1C* in IUGR Placentae

**FIGURE 2:**
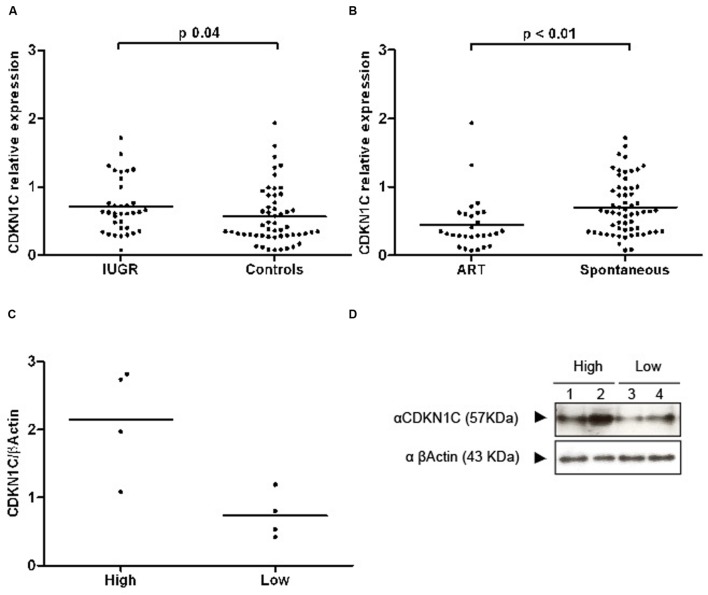
**Scatter plots representing RQ (relative quantification) values of *CDKN1C* transcripts and proteins. (A)** A graph showing the transcript abundance of *CDKN1C* in IUGR and control placenta samples with the line representing the median. **(B)** The expression profile in ART and spontaneously conceived samples. **(C)** Quantification of protein expression differences in lowly and highly transcribed placenta samples. **(D)** An example of a western blot used for protein quantification.

Expression levels of *CDKN1C* relative to the endogenous *RPL19* gene were 1.4 times higher in the IUGR group in a univariate analysis (IUGR RQ 0.77 ± 0.09 vs. controls 0.56 ± 0.06, Students’ *t p* = 0.04) (**Figure 2A**). For a better fitting in statistical analysis, RQ was transformed by Log10, rendering a distribution closer to normality. We found no correlation between *CDKN1C* and maternal age, height or weight or with gestational age or anthropometric parameters of the baby, suggesting that the relationship between expression and growth is specific to an effect of IUGR rather than based on birth weight (a full list of non-significant variables can be found in **Supplementary Table S3**).

Expression of *CDKN1C* was also found to be decreased in the placenta of primiparous women (log10 *CDKN1C*-1.40 ± 0.33 in primiparous vs. –1.14 ± 0.28 in multiparous, *p* < 0.001) and in gestations conceived by assisted reproduction (log10 *CDKN1C*-1.47 ± 0.35 in TRA vs. –1.22 ± 0.30 in spontaneous conceptions, *p* < 0.001). There were no differences by gender of the fetus, presence of labor, complication by pre-eclampsia, maternal smoking habits or between singleton and multiple pregnancies.

Multivariate analysis by direct logistic regression (dependent variable: IUGR yes/no) was performed to rule out the effect of confounding variables with an effect on *CDKN1C* expression in our sample (primiparity and ART); we also included pre-eclampsia as a covariate, as it was the only clinical feature with a different prevalence between the IUGR and control groups (prevalence of 22.2% vs. 3.8%, Chi-square test *p* = 0.01). Primiparity and ART were finally excluded from the model, as they did not have a statistically significant contribution. The final statistical model (Nagelkerke *R*^2^ 0.172, *p* = 0.002) included expression of *CDKN1C* and presence of preeclampsia. The effect of each of these variables on IUGR was significant [Log10 *CDKN1C* expression, Exp (B) 4.91, 95% CI 1.09–22.10, *p* = 0.038; preeclampsia, Exp (B) 5.78, 95% CI 1.12–29.73, *p* = 0.05], indicating an independent effect of each of these factors on IUGR.

Western blot analysis revealed that extreme mRNA levels of *CDKN1C* were also observed at the protein level with higher levels detected in IUGR placentae (**Figures 2C,D**). However, the difference at the protein level is smaller suggesting there is post-transcriptional regulation of *CDKN1C* in human placenta.

#### Expression Differences in ART Placenta Biopsies

Altered levels of expression of imprinted genes have been reported in both mouse and human placentas after the use of assisted reproductive technologies (ART; Katari et al., 2009; Iglesias-Platas et al., 2014) and placental epigenetic profiles seem particularly vulnerable (Fortier et al., 2008). Levels of expression of *CDKN1C* were lower in placentas of babies conceived by ART (**Figure 2B**). In our sample, mothers in the ART group were significantly older (35.5 ± 6.3 vs. 31.9 ± 5.8 years, Student’s *t p* = 0.01) and taller (166.7 ± 6.4 vs. 162.0 ± 7.2 cm, Student’s *t p* = 0.01), more likely to be primiparous (89.3% vs. 52.5%, Chi square *p* = 0.001), to carry a multiple pregnancy (78.6% vs. 16.4%, Chi square *p* < 0.001) and to be delivered by elective cesarean section, without having undergone labor (67.9% vs. 32.8%, Chi square *p* = 0.02). Gestational age at birth was lower in the ART group (32.4 ± 7.5 vs. 35.4 ± 4.8 weeks, Student’s *t p* = 0.04) and baby size was concordantly smaller, but there were no differences regarding anthropometry for gestational age. The groups were similar regarding gender of the newborn and incidence of IUGR and pre-eclampsia. To further explore the relationship between ART, expression of *CDKN1C* and the aforementioned clinical variables, we used hierarchical multiple regression, controlling for the identified confounders. The model including ART, maternal age and height, primiparity, labor and multiple gestation was borderline significant (adjusted *R*^2^ 0.112, *p* = 0.05) and ART and multiparity were the only statistically significant contributors to the variance of *CDKN1C* levels. A linear regression model for the variance of *CDKN1C* expression including only ART and primiparity had an adjusted *R*^2^ of 0.174 (*p* < 0.001), with both clinical values having a significant contribution (ART standardized β = 0.249, *p* = 0.01, primiparity standardized β = 0.284, *p* = 0.01).

#### Increased Expression in IUGR Despite Maintained Imprinted Expression

**FIGURE 3:**
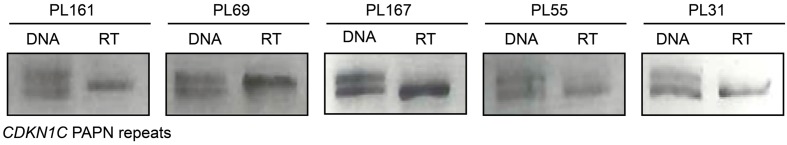
**Allele specific expression of the *CDKN1C* gene in placenta biopsies.** The first lane for each sample represents amplification of the PAPA repeat in genomic DNA. PCR products were visualized on 4% agarose gels and alleles discriminated based on amplicon size. Only heterozygous genotypes were selected for allelic expression analysis. The second lane is amplification from cDNA following reverse transcription showing monoallelic expression.

To ensure that *CDKN1C* maintained monoallelic expression, with transcription solely from the maternal allele, we determined the allelic origin of transcription in all heterozygous individuals. In total 10 samples were heterozygous for the PAPA repeat in exon 2. This represented all groups of gestational age, intrauterine growth and mode of conception. Monoallelic expression was observed in all cases (**Figure 3**), and origin was confirmed as maternal in three cases with homozygous mothers.

### Normal Methylation of *CDKN1C* in IUGR Placental Samples

**FIGURE 4:**
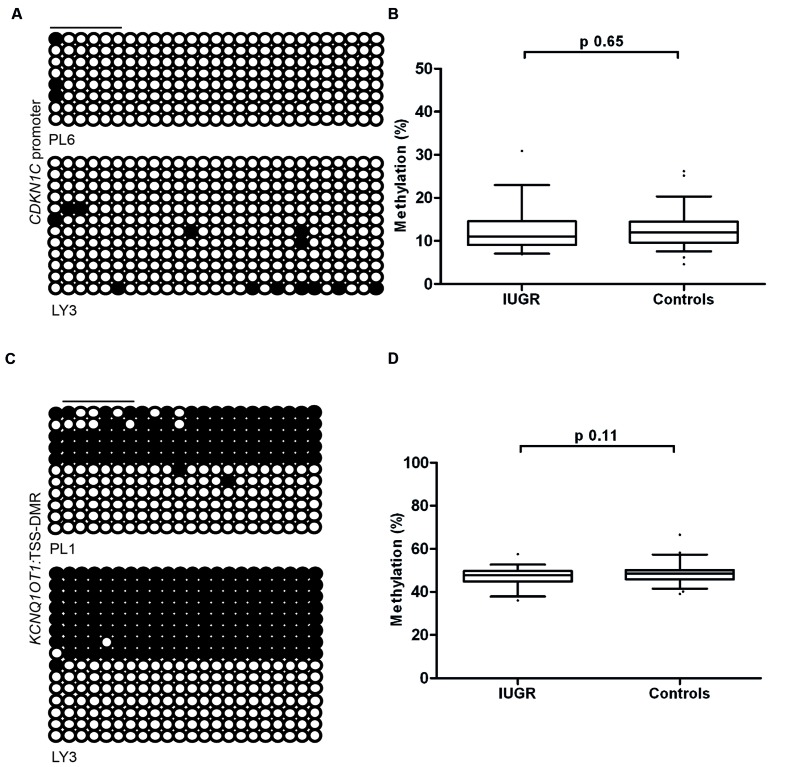
**The methylation profiles of the *CDKN1C* promoter and *KCNQ1OT1*:TSS-DMR. (A)** The left panels show an example of the placental and leukocyte methylation profiles of the 31 CpG dinucleotides analyzed within the *CDKN1C* promoter CpG island as determined by cloning and direct sequencing. **(B)** The methylation profiles, representing the average methylation of the six CpGs assayed by pyrosequencing within the *CDKN1C* promoter. **(C)** The bisulfite PCR profiling for the 22 CpG dinucleotides analyzed in the *KCNQ1OT1:*TSS-DMR and **(D)** the resulting methylation profile as determined by pyrosequencing. Each circle represents a CpG on the strand, and filled circles and open circles indicate methylated and unmethylated sites, respectively. Horizontal lines indicate the CpG sites analyzed by pyrosequencing. PL, placenta; LY, lymphocyte.

To determine if the differences in expression observed in IUGR and following ART is due to changes in DNA methylation at the *CDKN1C* promoter we performed pyrosequencing to quantify methylation levels. The bisulfite PCR product incorporated 31 CpG dinucleotides within the amplicon (**Figure 4A**), with the subsequent pyrosequencing analysis limited to six CpG dinucleotides due to sequence restrictions for the internal sequence primer and the limited length of the sequence reads. The arithmetic mean of the six CpG dinucleotides was used as a representative measure of the level of methylation for each placenta sample in our cohort. Pyrosequencing did not detect any differences in methylation between control and IUGR placentae (mean methylation 12.55 ± 0.53% in controls vs. 12.13 ± 0.79% in IUGR, Student’s *t p* = 0.65; **Figure 4B**) or between ART and spontaneously conceived pregnancies (mean methylation 11.21 ± 0.60% in ART vs. 12.86 ± 0.56% in spontaneous, Student’s *t p* = 0.09).

Since the maternal expression of *CDKN1C* is regulated in-*cis* by the *KCNQ1OT1*:TSS-DMR we also quantified the methylation of this region by pyrosequencing. We observed no deviation from the expected ∼50% methylation in any sample. This suggests that methylation defects at this imprinted DMR are not responsible for the variation in expression levels (**Figures 4C,D**). This is in agreement with other studies that have shown that changes in imprinted gene abundance in placenta samples are not attributable to methylation defects (Ishida et al., 2012; Iglesias-Platas et al., 2014).

### No Evidence for Genetic Variability Influencing *CDKN1C* Expression

Since the increased expression of *CDKN1C* in IUGR babies occurs without loss of imprinting, we hypothesized that underlying genetic variants may influence expression in a similar manner as previously reported for the nearby imprinted gene *PHLDA2 (*Ishida et al., 2012). We sequenced ∼1.2 kb upstream from the transcription start site, overlapping the promoter CpG island in two PCR products to identify polymorphisms. The UCSC genome browser (Build GRCh37/hg19) listed five SNPs in this region found in >1% of samples, encompassing rs116430081 to rs431222. However, upon sequence analysis all SNPs were identified in control and IUGR samples with similar frequencies as reported in dbSNP database^2^ (**Table 4**).

**Table 4:** SNP reference numbers, genetic variants, and population frequencies listed according to NCBI dbSNP database.

	SNP ID	Controls		IUGR		dbSNP 142	
Enhancer 3	rs11823023	G: 84%	A: 16%	G: 81%	A: 19%	G: 85%	A: 15%
	rs179432	A: 77%	G: 23%	A: 76%	G: 24%	A: 70%	G: 30%
	rs179433	C: 77%	T: 23%	C: 76%	T: 24%	C: 70%	T: 30%
	rs179434	A: 68%	C: 32%	A: 67%	C: 33%	A: 57%	C: 43%
	rs179435	A: 78%	G: 22%	A: 76%	G: 24%	A: 67%	G: 33%
	rs179436	G: 84%	A: 16%	G: 82%	A: 18%	G: 83%	A: 17%
Enhancer 2	rs2237884	T: 74%	C: 26%	T: 73%	C: 27%	T: 67%	C: 33%
	rs233434	G: 91%	A: 9%	G: 86%	A: 14%	G: 92%	A: 8%
	rs5789271	-: 56%	G: 44%	-: 60%	G: 40%	-:57 %	G: 43%
	rs12794000	C: 82%	T: 18%	C: 84%	T: 16%	C: 84%	T: 16%
Enhancer 1	rs202159835	-: 94%	+: 6%	-: 88%	+: 13%	-: 99%	+: 1%
	rs233451	G: 78%	A: 22%	G: 77%	A: 23%	G: 83%	A: 17%
	rs163184	C: 58%	T: 42%	T: 71%	T: 29%	C: 63%	T: 37%
Promoter	rs431222	C: 71%	T: 29%	C: 72%	T: 28%	C: 79%	T: 21%
	rs452338	G: 71%	T: 29%	G: 72%	T: 28%	G: 79%	T: 21%
	rs34738237	-: 66%	CA: 34%	-: 74%	CA: 26%	-: 74%	CA: 26%
	rs116430081	C: 100%	T: 0%	C: 99%	T: 1%	C: 99%	T: 1%

*The frequency of observed genetic variants in our cohort classified as control or IUGR.*

### Identifying Long-Range Enhancer Elements for *CDKN1C*

**FIGURE 5:**
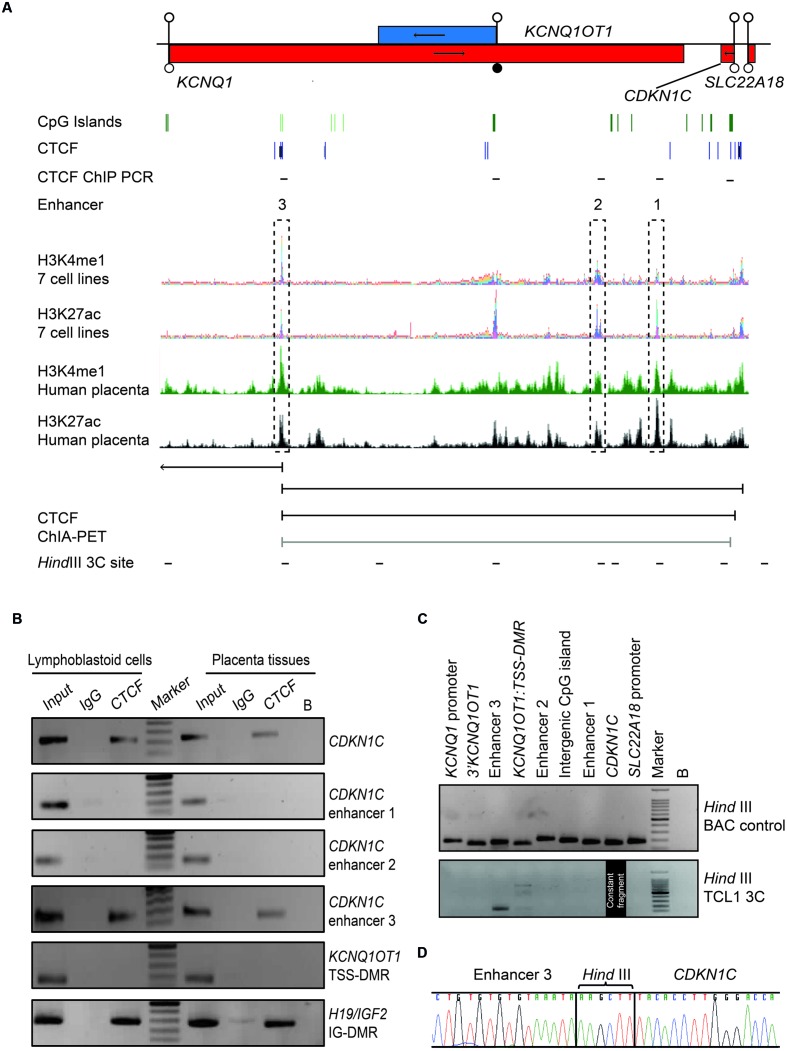
**Chromatin conformation capture (3C) interaction across the 440 kb *KCNQ1-CDKN1C* region in human placenta. (A)** Map of *KCNQ1OT1*-*CDKN1C* domain showing location of three putative enhancer regions in multiple cell types defined by overlapping H3K4me1 and H3K27ac ChIP-seq peaks and the relative position of transcripts, promoter and DMRs. The same intervals also show the canonical histone enhancer signature in first trimester placenta ChIP-seq datasets. ChIA-PET color coding represents signal enrichment based on aligned reads; Black for high frequency interactions, gray for medium intensity interactions. The positions of CTCF sites and HindIII sites utilized for 3C analysis are shown. **(B)** CTCF ChIP performed in placenta and lymphoblastoid cells. Input chromatin was used as a positive control (input 5%). ChIP-recovered DNA, analyzed using PCR and the resulting amplicons visualized using agarose gel electrophoresis. **(C)** All 3C PCR products were sized and visualized on a 2% agarose gels. The upper panel depicts the 3C PCR control in HindIII digested and ligated BAC control DNA. Each target primer combination was analyzed with the reverse primer of the constant region mapping to a HindIII site within the *CDKN1C* gene. The lower panel shows the 3C analysis performed on DNA-derived from cross-linked, digested and ligated chromatin from the placenta cell line TCL1. 3C PCR products of correct size were observed for the enhancer 3 interaction. **(D)** Sequence analysis reveals the appropriate ligation products following 3C PCR depicting higher-order chromatin contact between the *CDKN1C* constant fragment and the enhancer 3 region.

Both mouse transgenic studies and data obtained from rare patients with deletions/duplications have suggested that *cis*-acting regulatory elements are required for *Cdkn1c/CDKN1C* expression, with murine placenta-specific enhancer(s) located >315 kb from the gene (John et al., 2001). Recently, a bioinformatics search for conserved non-coding regions harboring the constitutive enhancer histone signature of H3K4me1, H3K27ac, and DNAse1 hypersensitivity sites (Heintzman et al., 2007; Creyghton et al., 2010; Encode Project Consortium, 2012) revealed three likely candidate regions (Cerrato et al., 2014) (**Figure 5A**). To identify potential *CDKN1C* enhancers in the human placenta we performed a bioinformatics search for co-localization of H3K4me1 and H3K27ac peaks in datasets generated from fetal placenta tissue available in the GEO data repository (GSM110284; GSM1102795). The ChIP-seq peaks were visualized using the UCSC genome browser ENCODE analysis Hub option. This analysis identified the same three intervals described by Cerrato et al. (2014) using ENCODE ChIP-seq data generated in somatic tissues and cell lines.

### Evidence for CTCF Mediated Chromatin Looping between Enhancers and *CDKN1C*

Recent studies have revealed that CTCF occupancy mediates intra- and interchromosomal contacts (Holwerda and de Laat, 2013). Several canonical CTCF binding sites within the *CDKN1C* gene body and proposed enhancer regions were identified using an *in silico* analysis of ENCODE datasets in multiple tissues (**Figure 5A**). To confirm *in vitro* binding we performed ChIP with CTCF antisera on normal lymphoblast cells and placenta (**Figure 5B**). The efficiency of the ChIP was confirmed by precipitation of the *H19/IGF2*:IG-DMR and no enrichment of the *KCNQ1OT1*:TSS-DMR. Subsequent analysis revealed precipitation of *CDKN1C* and enhancer 3 in both cell types, but not at enhancers 1 and 2, respectively.

Having confirmed CTCF binding at discrete locations within the *CDKN1C* locus we hypothesize that CTCF dimerization may orchestrate higher order chromatin loops (Rao et al., 2014; de Wit et al., 2015). Next, we interrogated publically available genome-wide ChIA-PET datasets which revealed that the CTCF site within the *CDKN1C* gene body physically interacts with the CTCF site with enhancer 3 located ∼360 kb away in both breast (MCF7) and blood (K562) cell lines, indicative of a constitutive chromatin loop (**Figure 5A**). To confirm this physical interaction we performed chromatin conformation capture experiments (3C) to identify potential chromatin folding. 3C-PCR assays were performed on the placental TCL1 cell line and interaction frequencies were determined between a constant HindIII site located near the CTCF binding site within *CDKN1C* and other HindIII sites throughout the locus. We identified strong interaction between the *CDKN1C* constant fragment with the CTCF site in the enhancer region 3 (**Figure 5C**). Direct sequencing of the PCR product revealed that the appropriate chimeric products result from the 3C ligations (**Figure 5D**). Unfortunately no informative SNPs were identified within the vicinity of the HindIII sites associated with these loops that would allow us to determine the parental origin of the resulting chemical products. These results suggest that the *CDKN1C* expression, at least in placenta, is dependent upon the active enhancer configuration and higher-order chromatin looping.

### Determining Genetic and Methylation Variation at Enhancers

**FIGURE 6:**
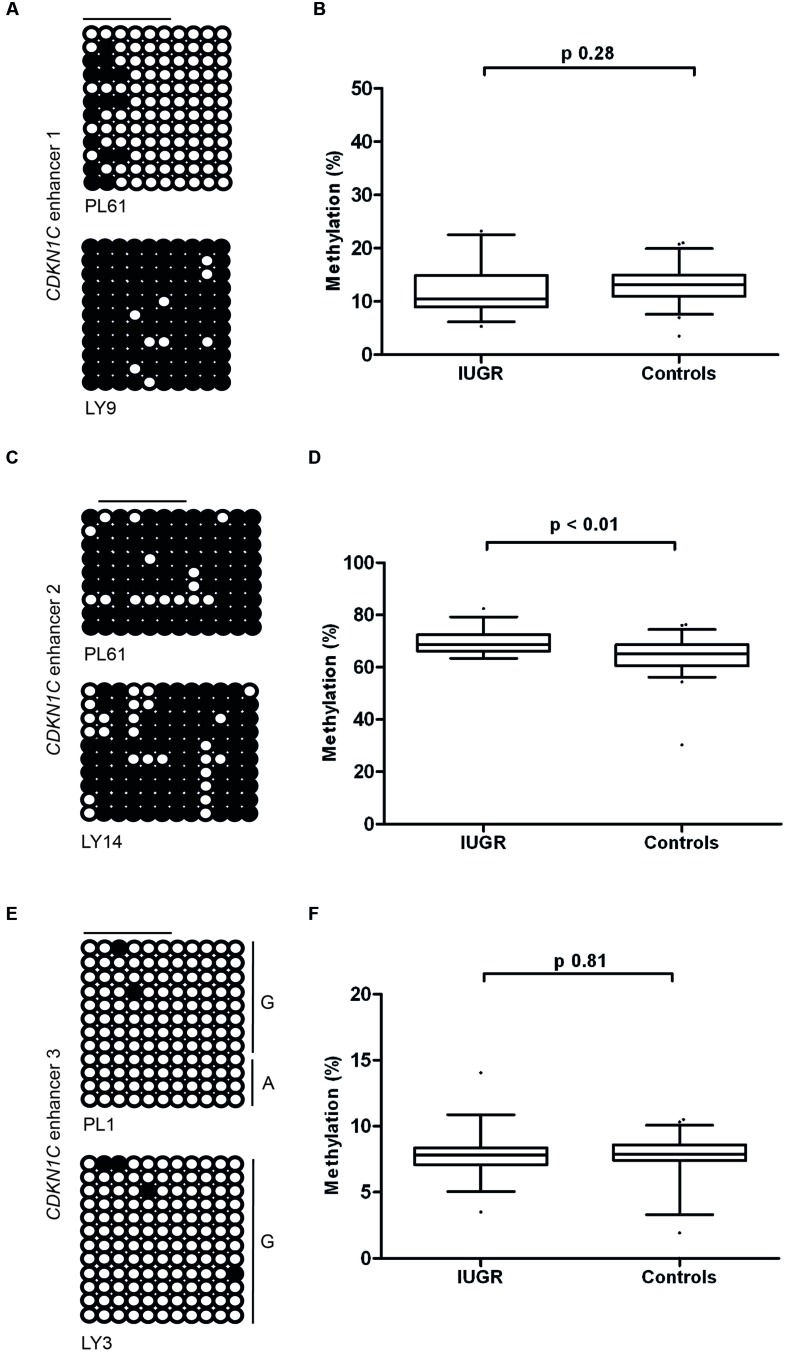
**Methylation at the three putative *CDKN1C* enhancer regions. (A,C,E)** The figures represent bisulfite PCR and sequencing for placenta and leukocytes samples where each row corresponds to an individual sequenced DNA clone. Each circle represents a CpG on the strand, and filled circles and open circles indicate methylated and unmethylated sites, respectively. Horizontal lines indicate the CpG sites analyzed by pyrosequencing. **(B,D,F)** The methylation profiles, representing the average methylation of assayed CpGs as determined by pyrosequencing in IUGR and control control placenta samples. PL, placenta; LY, lymphocyte.

Having identified the putative enhancer regions that may influence the expression of *CDKN1C*, we addressed if epigenetic or genetic variation within these regions were associated with IUGR in our samples. We optimized bisulfite pyrosequencing to quantify DNA methylation of multiple CpG dinucleotides within the enhancer regions. The CTCF sites associated with the long-range enhancer 3 are unmethylated in all control samples and we failed to detect any methylation changes with the IUGR profiles being within the control methylation ranges (mean methylation 7.75% ± 0.23% in controls and 7.83% ± 0.26% in IUGR, Student’s *t p* = 0.81). Similarly, despite tissue-specific differences observed between placenta and leukocytes, we failed to identify methylation changes at enhancer 1 associated with IUGR (mean methylation 13.15% ± 0.46% in controls and 12.2% ± 0.84% in IUGR, Student’s *t p* = 0.28). However, a small, but statistically significant difference between normal and IUGR placentas was observed at enhancer 2 (mean methylation 64.53% ± 0.92% in controls and 69.48% ± 0.77% in IUGR, Student’s *t p* < 0.001; **Figure 6**). Furthermore, genotype analysis of polymorphisms within the enhancer regions failed to reveal any haplotypes enriched in the IUGR group (**Table 4**). This suggests that large variation within the enhancer intervals do not account for the difference in *CDKN1C* expression observed in IUGR.

## Discussion

Much of the epigenetic research associated with IUGR has focused on imprinted loci since they are critical for early growth and metabolic adaptation; however, most data, including our previous studies (Camprubí et al., 2013; Iglesias-Platas et al., 2014), have revealed that methylation fluctuates very little at these loci. This is presumably due to the fact that most imprinted DMRs are associated with multiple layers of epigenetic regulation (McEwen and Ferguson-Smith, 2010) that tightly maintain gene expression in the face of any environmental perturbation like maternal undernutrition (Radford et al., 2012). In future studies it would be interesting to determine if changes in histone-tail modifications, including lysine methylation, influence expression in samples with normal DNA methylation.

The data presented in this study highlights the role of the imprinted *CDKN1C* transcript in fetal growth via a placenta-mediated mechanism. We have shown increased *CDKN1C* expression in IUGR placentas after adjustment for other variables influencing size at birth. The precise mechanism of action of *CDKN1C* in the human placenta is unknown, but is likely to involve regulation of the cell cycle. It would be interesting to determine if the aberrant growth associated with higher expression levels of *CDKN1C* continues following delivery, or if there is catch-up growth indicative of placenta dysfunction. Constitutional growth restriction associated with both IMAGe syndrome and SRS have been reported in rare patients with gain-of-function mutations in the *CDKN1C* PCNA domain that presumably alters protein clearance by the ubiquitin-proteasome. Whilst, we do not see mutations of this domain in non-syndromic IUGR (data not shown), we do observe increased protein abundance in IUGR placenta samples. Interestingly the magnitude of increased mRNA expression and protein levels differs greatly, suggesting there is extensive post-transcriptional regulation of *CKDN1C*. Recently, the microRNA miR-221 has been shown to regulate *CDKN1C* mRNA levels (Sun et al., 2011) and this specific miRNA has been shown to be plentiful in third trimester/term placentas (Gu et al., 2013).

Imprinted gene expression in the placenta can be influenced by many factors including differences in the sampling site, mode of delivery (Janssen et al., 2015), fetal sex (Iglesias-Platas et al., 2014), and gestational age (Demetriou et al., 2014). Regional variation in the expression of *PHLDA2* has previously been reported with modest elevation of expression in samples taken at the distal edge of the placenta compared to the ones near the cord insertion site (Janssen et al., 2015) which may partially be attributed to differences in placental architecture and blood supply. To overcome this we routinely obtained biopsies from the same location on the fetal side of the placenta within 2 cm of the cord insertion. In addition the same authors observed elevated *CDKN1C* expression in placenta from labored deliveries compared with elective cesarean sections. We do not observe any differences in our larger cohort of samples.

Furthermore difference in expression have can also be modulated by gestational age, with the transcript levels of the *PHLDA2* gene showing an association with birth weight (although not IUGR) in term samples (Apostolidou et al., 2007; Ishida et al., 2012) but not in first trimester chorionic villus sampling (Demetriou et al., 2014). Similar gestational age associations have also been observed for additional imprinted genes, including *GRB10* in first trimester placenta samples and *IGF2* in a series of normal term biopsies (Moore et al., 2015) but it has not been established if differences occur in biopsies from complicated pregnancies. Interestingly different studies have reported conflicting results for *CDKN1C* influencing fetal size with three reports describing a negative correlation with birth weight (McMinn et al., 2006; Moore et al., 2015; Piyasena et al., 2015). It will be important to verify if changes in imprinted gene expression are a direct cause of pregnancy complications, or alternatively, they may reflect a common adaptive compensatory mechanism in the placenta.

The increased abundance of *CDKN1C*, we observe in IUGR placentas was not paralleled by changes in methylation at the *CDKN1C* promoter or *KCNQ1OT1*:TSS*-*DMR. Other authors have also found expression levels differences in imprinted genes without obvious methylation changes in the corresponding imprinted DMRs. This presumably reflects transcriptional deregulation by other *trans*-acting mechanisms, such as transcription factor binding (McMinn et al., 2006; Ishida et al., 2012; Cordeiro et al., 2014; Iglesias-Platas et al., 2014; Piyasena et al., 2015). We have previously shown that the expression levels of *PLAGL1* correlated with *CDKN1C* levels, with *PLAGL1* presumably exerting its influence as a zinc finger transcription factor (Iglesias-Platas et al., 2014). However, the precise location of the binding sites are unknown since the long-range enhancer elements have yet to be characterized. Recently a bioinformatics screen for somatic enhancers identified three candidate regions (Cerrato et al., 2014) that are the same as we identified in placenta. Experiments performed by John et al. (2001) using BAC transgenes indicate that, in addition to the DMR within the promoter of *Kcnq1ot1*, expression of *Cdkn1c* requires interaction with a distant tissue-specific enhancer located within the *Kcnq1* gene. Using such an approach, the enhancers for expression in the placenta were not identified, suggesting they might be located more than 315 kb from *Cdkn1c*. This is consistent with our observation as one of the putative enhancers we identify (region 3) maps ∼360 kb from *CDKN1C* and we show they physically interact in placenta.

We have previously demonstrated that the imprinted domains associated with *PLAGL1* (Iglesias-Platas et al., 2014) and *PEG13/KCNK9* (Court et al., 2014a) require additional transcription factors or active enhancers to facilitate imprinted transcription from constitutive CTCF-mediated chromatin loops that are stable and independent of transcription. Similarly the data we present here suggest that CTCF orchestrates biallelic higher-order chromatin interactions within the *KCNQ1*-domain since the binding sites for CTCF, a known methylation-sensitive DNA binding protein responsible for the looping, are all unmethylated. Interestingly, we do not observe CTCF binding within the *KCNQ1OT1*:TSS-DMR, suggesting that this interval does not act as a methylation-sensitive insulator as has been observed in mice (Du et al., 2003; Shin et al., 2008). However, prominent CTCF binding was observed 5 kb downstream of the DMR in most tissues analyzed by ENCODE. We hypothesize that the level of *CDKN1C* transcription is regulated by a combination of the shared enhancers and endogenous promoter sequences, with the latter conferring the allelic specificity due to the accumulation of repressive histone modification on the paternal allele (Monk et al., 2006).

## Conclusion

Our results show DNA methylation independent differences of *CDKN1C* expression in placenta in non-syndromic IUGR and between pregnancies of primiparous versus multiparous mothers and gestations conceived spontaneously or by assisted reproduction. Our results support the idea that distant enhancers physically interact via long-range chromatin looping which in turn regulate *CDKN1C* expression. Deciphering the role of these putative enhancer elements in regulating tissue-specific expression of *CDKN1C* will be important to understand the molecular etiologies of non-syndromic IUGR, SRS, BWS, and IMAGe syndrome.

## Author Contributions

Conceived and designed the experiments: II-P and DM. Performed the experiments: ML-A, II-P, and DM. Analyzed the data: ML-A, II-P, and DM. Wrote the paper: DM. Discussed and critically edited the manuscript: ML-A, II-P, and DM.

## Conflict of Interest Statement

The authors declare that the research was conducted in the absence of any commercial or financial relationships that could be construed as a potential conflict of interest.

## Footnotes

1 http://www.imprinting-disorders.eu

2 http://www.ncbi.nlm.nih.gov/snp

## Supplementary Material

Table S1 **Oligonucleotide PCR primers used in this study**.

Table S2 **Summary of the experimental results that based our selection for internal control genes in placental tissue.** *MRPL19*, mitochondrial ribosomal transcript; GUS3, glucuronidase 3; *HPRT*, hypoxanthine guanine phosphoribosyl transferase; *NCL*, nucleolin; *GAPDH*, glyceraldehyde-3-phosphate dehydrogenase; *MRLPL19* showed the smallest variation between placental samples and the closest amplification characteristics (cycle threshold, *C*_T_) to those of our genes of interest.

Table S3 **A summary of non-significant variables that are not associated with changes in *CDKN1C* expression levels**.

## References

[B1] Andrews S. C. Wood M. D. Tunster S. J. Barton S. C. Surani M. A. John R. M. (2007). Cdkn1c (p57Kip2) is the major regulator of embryonic growth within its imprinted domain on mouse distal chromosome 7. *BMC Dev. Biol.* 7:53 10.1186/1471-213X-7-53 PMC1891291 17517131

[B2] Apostolidou S. Abu-Amero S. O’Donoghue K. Frost J. Olafsdottir O. Chavele K. M. (2007). Elevated placental expression of the imprinted PHLDA2 gene is associated with low birth weight. *J. Mol. Med. (Berl.)* 85 379–387. 10.1007/s00109-006-0131-8 17180344

[B3] Arboleda V. A. Lee H. Parnaik R. Fleming A. Banerjee A. Ferraz-de-Souza B. (2012). Mutations in the PCNA-binding domain of CDKN1C cause IMAGe syndrome. *Nat. Genet.* 44 788–792. 10.1038/ng.2275 22634751 PMC3386373

[B4] Begemann M. Spengler S. Gogiel M. Grasshoff U. Bonin M. Betz R. C. (2012). Clinical significance of copy number variations in the 11p15.5 imprinting control regions: new cases and review of the literature. *J. Med. Genet.* 49 547–553. 10.1136/jmedgenet-2012-100967 22844132 PMC3439641

[B5] Brioude F. Oliver-Petit I. Blaise A. Praz F. Rossignol S. Le Jule M. (2013). CDKN1C mutation affecting the PCNA-binding domain as a cause of familial Russell Silver syndrome. *J. Med. Genet.* 50 823–830. 10.1136/jmedgenet-2013-101691 24065356

[B6] Camprubí C. Iglesias-Platas I. Martin-Trujillo A. Salvador-Alarcon C. Rodriguez M. A. Barredo D. R. (2013). Stability of genomic imprinting and gestational-age dynamic methylation in complicated pregnancies conceived following assisted reproductive technologies. *Biol. Reprod.* 89 50 10.1095/biolreprod.113.108456 23884645

[B7] Carter A. M. Enders A. C. (2004). Comparative aspects of trophoblast development and placentation. *Reprod. Biol. Endocrinol.* 5 46 10.1186/1477-7827-2-46 PMC455692 15236656

[B8] Caspary T. Cleary M. A. Baker C. C. Guan X. J. Tilghman S. M. (1998). Multiple mechanisms regulate imprinting of the mouse distal chromosome 7 gene cluster. *Mol. Cell. Biol.* 18 3466–3474. 10.1128/MCB.18.6.3466 9584186 PMC108927

[B9] Cerrato F. De Crescenzo A. Riccio A. (2014). Looking for CDKN1C enhancers. *Eur. J. Hum. Genet.* 22 442–443. 10.1038/ejhg.2013.234 24129436 PMC3953923

[B10] Cordeiro A. Neto A. P. Carvalho F. Ramalho C. Dória S. (2014). Relevance of genomic imprinting in intrauterine human growth expression of CDKN1C, H19, IGF2, KCNQ1 and PHLDA2 imprinted genes. *J. Assist. Reprod. Genet.* 31 1361–1368. 10.1007/s10815-014-0278-0 24986528 PMC4171407

[B11] Court F. Camprubi C. Garcia C. V. Guillaumet-Adkins A. Sparago A. Seruggia D. (2014a). The PEG13-DMR and brain-specific enhancers dictate imprinted expression within the 8q24 intellectual disability risk locus. *Epigenetics Chromatin* 7 5 10.1186/1756-8935-7-5 PMC3986935 24667089

[B12] Court F. Tayama C. Romanelli V. Martin-Trujillo A. Iglesias-Platas I. Okamura K. (2014b). Genome-wide parent-of-origin DNA methylation analysis reveals the intricacies of human imprinting and suggests a germline methylation-independent mechanism of establishment. *Genome Res.* 24 554–569. 10.1101/gr.164913.113 24402520 PMC3975056

[B13] Creyghton M. P. Cheng A. W. Welstead G. G. Kooistra T. Carey B. W. Steine E. J. (2010). Histone H3K27ac separates active from poised enhancers and predicts developmental state. *Proc. Natl. Acad. Sci. U.S.A.* 107 21931–21936. 10.1073/pnas.1016071107 21106759 PMC3003124

[B14] de Wit E. Vos E. S. Holwerda S. J. Valdes-Quezada C. Verstegen M. J. Teunissen H. (2015). CTCF binding polarity determines chromatin looping. *Mol. Cell* 60 676–684. 10.1016/j.molcel.2015.09.023 26527277

[B15] Demetriou C. Abu-Amero S. Thomas A. C. Ishida M. Aggarwal R. Al-Olabi L. (2014). Paternally expressed, imprinted insulin-like growth factor-2 in chorionic villi correlates significantly with birth weight. *PLoS ONE* 9:e85454 10.1371/journal.pone.0085454 PMC3893199 24454871

[B16] Diaz-Meyer N. Day C. D. Khatod K. Maher E. R. Cooper W. Reik W. (2003). Silencing of CDKN1C (p57KIP2) is associated with hypomethylation at KvDMR1 in Beckwith-Wiedemann syndrome. *J. Med. Genet.* 40 797–801. 10.1136/jmg.40.11.797 14627666 PMC1735305

[B17] Du M. Beatty L. G. Zhou W. Lew J. Schoenherr C. Weksberg R. (2003). Insulator, and silencer sequences in the imprinted region of human chromosome 11p15. 5. *Hum. Mol. Genet.* 12 1927–1939. 10.1093/hmg/ddg194 12874112

[B18] Eggermann T. Algar E. Lapunzina P. Mackay D. Maher E. R. Mannens M. (2014). Clinical utility gene card for: Beckwith–Wiedemann syndrome. *Eur. J. Hum. Genet.* 22. 10.1038/ejhg.2013.132 PMC3925261 23820480

[B19] Eggermann T. Perez de Nanclares G. Maher E. R. Temple I. K. Tümer Z. Monk D. (2015). Imprinting disorders: a group of congenital disorders with overlapping patterns of molecular changes affecting imprinted loci. *Clin. Epigenetics* 7 123 10.1186/s13148-015-0143-8 PMC4650860 26583054

[B20] Encode Project Consortium (2012). An integrated encyclopedia of DNA elements in the human genome. *Nature* 489 57–74. 10.1038/nature11247 22955616 PMC3439153

[B21] Ferguson-Smith A. C. (2011). Genomic imprinting: the emergence of an epigenetic paradigm. *Nat. Rev. Genet.* 12 565–575. 10.1038/nrg3032 21765458

[B22] Fortier A. L. Lopes F. L. Darricarre‘re N. Martel J. Trasler J. M. (2008). Superovulation alters the expression of imprinted genes in the midgestation mouse placenta. *Hum. Mol. Genet.* 17 1653–1665. 10.1093/hmg/ddn055 18287259

[B23] Gu Y. Sun J. Groome L. J. Wang Y. (2013). Differential miRNA expression profiles between the first and third trimester human placentas. *Am. J. Physiol. Endocrinol. Metab.* 304 E836–E843. 10.1152/ajpendo.00660.2012 23443922 PMC3625781

[B24] Hamajima N. Johmura Y. Suzuki S. Nakanishi M. Saitoh S. (2013). Increased protein stability of CDKN1C causes a gain-of-function phenotype in patients with IMAGe syndrome. *PLoS ONE* 8:e75137 10.1371/journal.pone.0075137 PMC3787065 24098681

[B25] Hatada I. Mukai T. (1995). Genomic imprinting of p57KIP2, a cyclin-dependent kinase inhibitor, in mouse. *Nat. Genet.* 11 204–206. 10.1038/ng1095-204 7550351

[B26] Heintzman N. D. Stuart R. K. Hon G. Fu Y. Ching C. W. Hawkins R. D. (2007). Distinct and predictive chromatin signatures of transcriptional promoters and enhancers in the human genome. *Nat. Genet.* 39 311–318. 10.1038/ng1966 17277777

[B27] Hiura H. Obata Y. Komiyama J. Shirai M. Kono T. (2006). Oocyte growth-dependent progression of maternal imprinting in mice. *Genes Cells* 11 353–361. 10.1111/j.1365-2443.2006.00943.x 16611239

[B28] Holwerda S. J. de Laat W. (2013). CTCF: the protein, the binding partners, the binding sites and their chromatin loops. *Philos. Trans. R. Soc. Lond. B Biol. Sci.* 368 20120369 10.1098/rstb.2012.0369 PMC3682731 23650640

[B29] Iglesias-Platas I. Martin-Trujillo A. Petazzi P. Guillaumet-Adkins A. Esteller M. Monk D. (2014). Altered expression of the imprinted transcription factor PLAGL1 deregulates a network of genes in the human IUGR placenta. *Hum. Mol. Genet.* 23 6275–6285. 10.1093/hmg/ddu347 24993786 PMC4334785

[B30] Ishida M. Monk D. Duncan A. J. Abu-Amero S. Chong J. Ring S. M. (2012). Maternal inheritance of a promoter variant in the imprinted PHLDA2 gene significantly increases birth weight. *Am. J. Hum. Genet.* 90 715–719. 10.1016/j.ajhg.2012.02.021 22444668 PMC3322226

[B31] Janssen A. B. Tunster S. J. Savory N. Holmes A. Beasley J. Parveen S. A. (2015). Placental expression of imprinted genes varies with sampling site and mode of delivery. *Placenta* 36 790–795. 10.1016/j.placenta.2015.06.011 26162698 PMC4535278

[B32] John R. M. Ainscough J. F. Barton S. C. Surani M. A. (2001). Distant cis-elements regulate imprinted expression of the mouse p57( Kip2) (Cdkn1c) gene: implications for the human disorder, Beckwith–Wiedemann syndrome. *Hum. Mol. Genet.* 10 1601–1609. 10.1093/hmg/10.15.1601 11468278

[B33] Katari S. Turan N. Bibikova M. Erinle O. Chalian R. Foster M. (2009). DNA methylation and gene expression differences in children conceived in vitro or in vivo. *Hum. Mol. Genet.* 18 3769–3778. 10.1093/hmg/ddp319 19605411 PMC2748887

[B34] Kirchmaier A. L. (2011). Ub-family modifications at the replication fork: regulating PCNA-interacting components. *FEBS Lett.* 585 2920–2928. 10.1016/j.febslet.2011.08.008 21846465

[B35] Kong A. Steinthorsdottir V. Masson G. Thorleifsson G. Sulem P. Besenbacher S. (2009). Parental origin of sequence variants associated with complex diseases. *Nature* 462 868–874. 10.1038/nature08625 20016592 PMC3746295

[B36] Lee M. H. Reynisdóttir I. Massagué J. (1995). Cloning of p57KIP2, a cyclin-dependent kinase inhibitor with unique domain structure and tissue distribution. *Genes Dev.* 9 639–649. 10.1101/gad.9.6.639 7729683

[B37] Lee M. P. Hu R. J. Johnson L. A. Feinberg A. P. (1997). Human KVLQT1 gene shows tissue-specific imprinting and encompasses Beckwith-Wiedemann syndrome chromosomal rearrangements. *Nat. Genet.* 15 181–185. 10.1038/ng0297-181 9020845

[B38] Lewis A. Mitsuya K. Umlauf D. Smith P. Dean W. Walter J. (2004). Imprinting on distal chromosome 7 in the placenta involves repressive histone methylation independent of DNA methylation. *Nat. Genet.* 36 1291–1295. 10.1038/ng1468 15516931

[B39] Lim D. H. Maher E. R. (2010). Genomic imprinting syndromes and cancer. *Adv. Genet.* 70 145–175. 10.1016/B978-0-12-380866-0.60006-X 20920748

[B40] Mancini-Dinardo D. Steele S. J. Levorse J. M. Ingram R. S. Tilghman S. M. (2006). Elongation of the Kcnq1ot1 transcript is required for genomic imprinting of neighboring genes. *Genes Dev.* 20 1268–1282. 10.1101/gad.1416906 16702402 PMC1472902

[B41] Matsuoka S. Edwards M. C. Bai C. Parker S. Zhang P. Baldini A. (1995). p57KIP2, a structurally distinct member of the p21CIP1 Cdk inhibitor family, is a candidate tumor suppressor gene. *Genes Dev.* 9 650–662. 10.1101/gad.9.6.650 7729684

[B42] McEwen K. R. Ferguson-Smith A. C. (2010). Distinguishing epigenetic marks of developmental and imprinting regulation. *Epigenetics Chromatin* 3 2 10.1186/1756-8935-3-2 PMC2841594 20180964

[B43] McMinn J. Wei M. Schupf N. Cusmai J. Johnson E. B. Smith A. C. (2006). Unbalanced placental expression of imprinted genes in human intrauterine growth restriction. *Placenta* 27 540–549. 10.1016/j.placenta.2005.07.004 16125225

[B44] Miller R. J. Sullivan M. C. Hawes K. Marks A. K. (2009). The effects of perinatal morbidity and environmental factors on health status of preterm children at age 12. *J. Pediatr. Nurs.* 24 101–114. 10.1016/j.pedn.2008.02.031 19268232 PMC2742999

[B45] Mohammad F. Pandey G. K. Mondal T. Enroth S. Redrup L. Gyllensten U. (2012). Long noncoding RNA-mediated maintenance of DNA methylation and transcriptional gene silencing. *Development* 139 2792–2803. 10.1242/dev.079566 22721776

[B46] Monk D. (2015). Genomic imprinting in the human placenta. *Am. J. Obstet. Gynecol.* 213(Suppl. 4), S152–S162. 10.1016/j.ajog.2015.06.032 26428495

[B47] Monk D. Arnaud P. Apostolidou S. Hills F. A. Kelsey G. Stanier P. (2006). Limited evolutionary conservation of imprinting in the human placenta. *Proc. Natl. Acad. Sci. U.S.A.* 103 6623–6628. 10.1073/pnas.0511031103 16614068 PMC1564202

[B48] Moore G. E. Ishida M. Demetriou C. Al-Olabi L. Leon L. J. Thomas A. C. (2015). The role and interaction of imprinted genes in human fetal growth. *Philos. Trans. R. Soc. Lond. B Biol. Sci.* 370 20140074 10.1098/rstb.2014.0074 PMC4305174 25602077

[B49] Okae H. Hiura H. Nishida Y. Funayama R. Tanaka S. Chiba H. (2012). Re-investigation and RNA sequencing-based identification of genes with placenta-specific imprinted expression. *Hum. Mol. Genet.* 21 548–558. 10.1093/hmg/ddr488 22025075

[B50] Piyasena C. Reynolds R. M. Khulan B. Seckl J. R. Menon G. Drake A. J. (2015). Placental 5-methylcytosine and 5-hydroxymethylcytosine patterns associate with size at birth. *Epigenetics* 10 692–697. 10.1080/15592294.2015.1062963 26091021 PMC4623028

[B51] Radford E. J. Isganaitis E. Jimenez-Chillaron J. Schroeder J. Molla M. Andrews S. (2012). An unbiased assessment of the role of imprinted genes in an intergenerational model of developmental programming. *PLoS Genet.* 8:e1002605 10.1371/journal.pgen.1002605 PMC3325178 22511876

[B52] Rao S. S. Huntley M. H. Durand N. C. Stamenova E. K. Bochkov I. D. Robinson J. T. (2014). A 3D map of the human genome at kilobase resolution reveals principles of chromatin looping. *Cell* 159 1665–1680. 10.1016/j.cell.2014.11.021 25497547 PMC5635824

[B53] Romero S. T. Geiersbach K. B. Paxton C. N. Rose N. C. Schisterman E. F. Branch D. W. (2015). Differentiation of genetic abnormalities in early pregnancy loss. *Ultrasound Obstet. Gynecol.* 45 89–94. 10.1002/uog.14713 25358469 PMC6157625

[B54] Sanchez-Delgado M. Martin-Trujillo A. Tayama C. Vidal E. Esteller M. Iglesias-Platas I. (2015). Absence of maternal methylation in biparental hydatidiform moles from women with NLRP7 maternal-effect mutations reveals widespread placenta-specific imprinting. *PLoS Genet.* 11:e1005644 10.1371/journal.pgen.1005644 PMC4636177 26544189

[B55] Schmitthen T. D. Livak K. J. (2008). Analysing real-time PCR data by the comparative C(T) method. *Nat. Protoc.* 3 1101–1108. 10.1038/nprot.2008.73 18546601

[B56] Shin J. Y. Fitzpatrick G. V. Higgins M. J. (2008). Two distinct mechanisms of silencing by the KvDMR1 imprinting control region. *EMBO J.* 27 168–178. 10.1038/sj.emboj.7601960 18079696 PMC2206141

[B57] Society for Maternal-Fetal Medicine Publications Committee Berkley Chauhan E. Abuhamad S. P. A. (2012). Doppler assessment of the fetus with intrauterine growth restriction. *Am. J. Obstet. Gynecol.* 206 300–308. 10.1016/j.ajog.2012.01.022 22464066

[B58] Sood R. Zehnder J. L. Druzin M. L. Brown P. O. (2006). Gene expression patterns in human placenta. *Proc. Natl. Acad. Sci. U.S.A.* 103 5478–5483. 10.1073/pnas.0508035103 16567644 PMC1414632

[B59] Sun K. Wang W. Zeng J. J. Wu C. T. Lei S. T. Li G. X. (2011). MicroRNA-221 inhibits CDKN1C/p57 expression in human colorectal carcinoma. *Acta Pharmacol. Sin.* 32 375–384. 10.1038/aps.2010.206 21278784 PMC4002764

[B60] Takahashi K. Nakayama K. Nakayama K. (2000). Mice lacking a CDK inhibitor, p57Kip2, exhibit skeletal abnormalities and growth retardation. *J. Biochem.* 127 73–83. 10.1093/oxfordjournals.jbchem.a022586 10731669

[B61] Thorvaldsen J. L. Duran K. L. Bartolomei M. S. (1998). Deletion of the H19 differentially methylated domain results in loss of imprinted expression of H19 and Igf2. *Genes Dev.* 12 3693–3702. 10.1101/gad.12.23.3693 9851976 PMC317260

[B62] Tokino T. Urano T. Furuhata T. Matsushima M. Miyatsu T. Sasaki S. (1996). Characterization of the human p57KIP2 gene: alternative splicing, insertion/deletion polymorphisms in VNTR sequences in the coding region, and mutational analysis. *Hum. Genet.* 97 625–631. 10.1007/BF02281873 8655143

[B63] Tunster S. J. Van de Pette M. John R. M. (2011). Fetal overgrowth in the Cdkn1c mouse model of Beckwith-Wiedemann syndrome. *Dis. Models Mech.* 4 814–821. 10.1242/dmm.007328 PMC3209650 21729874

[B64] Umlauf D. Goto Y. Cao R. Cerqueira F. Wagschal A. Zhang Y. (2004). Imprinting along the Kcnq1 domain on mouse chromosome 7 involves repressive histone methylation and recruitment of Polycomb group complexes. *Nat. Genet.* 36 1296–1300. 10.1038/ng1467 15516932

[B65] Watanabe H. Pan Z. Q. Schreiber-Agus N. DePinho R. A. Hurwitz J. Xiong Y. (1998). Suppression of cell transformation by the cyclin-dependent kinase inhibitor p57KIP2 requires binding to proliferating cell nuclear antigen. *Proc. Natl. Acad. Sci. U.S.A.* 95 1392–1397. 10.1073/pnas.95.4.1392 9465025 PMC19016

[B66] Westbury J. Watkins M. Ferguson-Smith A. C. Smith J. (2001). Dynamic temporal and spatial regulation of the cdk inhibitor p57(kip2) during embryo morphogenesis. *Mech. Dev.* 109 83–89. 10.1016/S0925-4773(01)00512-3 11677056

[B67] Yatsuki H. Joh K. Higashimoto K. Soejima H. Arai Y. Wang Y. (2002). Domain regulation of imprinting cluster in Kip2/Lit1 subdomain on mouse chromosome 7F4/F5: large-scale DNA methylation analysis reveals that DMR-Lit1 is a putative imprinting control region. *Genome Res.* 12 1860–1870. 10.1101/gr.110702 12466290 PMC187562

