# Supplementary material for: Epigenetic Characterization of CDKN1C in Placenta Samples from Non-syndromic Intrauterine Growth Restriction
Source: Front Genet. 2016 Apr 26;7:62. doi: 10.3389/fgene.2016.00062 (PMC4844605; doi:10.3389/fgene.2016.00062)
Supplement: Table S1 — Oligonucleotide PCR primers used in this study. [file Table_1.DOCX]

| **Primer name** | **Oligonucleotide sequence** |
| --- | --- |
| **Mutation/polymorphism screen** |  |
| CDKN1C PAPAn F  CDKN1C PAPAn R | AGGAGGCGCCGGAGCAGCTG  GCCCCTGGTTCGCGCCCTGC |
| PCNA exon 2 F  PCNA exon 2 R | CTCGCTGACCAGCTGCACTC  AAGCCGCTGGAGGGCACAACA |
| PCNA exon 3 F  PCNA exon 3 R | TGCGCGGCGACGTAAACAAAGCT  CCCCAGGTGCGCTGTACTC |
| CDKN1C promoter 1F  CDKN1C promoter 1R | TCCTTTGTCTGCAGGCGGGGGCCT  GACTCGAGGGCCTTAGGGCCAGCAGG |
| CDKN1C promoter 2F  CDKN1C promoter 2R | GTTGTACTGGGTAAAAGTGAAAG  TCTCCTGGGTTAACGCCCTGT |
| Enhancer 1 F  Enhancer 1 R | GGTGCTGGAAGAGCAAGCACCACG  TTGCCCTGCCTCTCTCTTTACT |
| Enhancer 1.1 F  Enhancer 1.1 R | GTCACGGTGGAAAGAGAGTCCAG  AGGACACTGAGCTGAGCAGGCTGA |
| Enhancer 2 F  Enhancer 2 R | TCCCGCTGCCTCTCGTAGTCT  GCAGTGTGGGCTCAGCCATGGG |
| Enhancer 2.1 F  Enhancer 2.1 R | TGGCTTTCTCTTTGCGCAAGGGC  CTCCTGCACGTCAGGGATGTTCAC |
| Enhancer 3 F  Enhancer 3 R | CCTCCAAGATGGTCCTCATCCC  AGTGGAAAAGGCAGAGGGGA |
| Enhancer 3.1 F  Enhancer 3.1 R | GCTCTGGGAGGCTCACAAGCATCTG  GTACATCCGCCTGTCAAGGCCAG |
| Enhancer 3.2 F  Enhancer 3.2 R | GGACGGCTGTTGGGTTCTTG  GCAGCTCCTGAGTTGCATGTAG |
| **Bisulphite PCRs** |  |
| *CDKN1C* promoter Bis F | GGTTTTTTTGTTTGTAGGYGGG |
| *CDKN1C* promoter Bis R(Bio) | AACCAACAAATATAAAAACTCCAA |
| *CDKN1C* promoter Seq F | GGGGGTTTTAGTTTAG |
| *CDKN1C* enhancer 3 Bis F | AGGGTTGTGGGGATTAGAGTATA |
| *CDKN1C* enhancer 3 Bis R (Bio) | CCCAAAACTATAATTTAAACTCCC |
| *CDKN1C* enhancer 3 Seq F | GAGGGAGGTTGTGTAGGGTTG |
| *CDKN1C* enhancer 2 Bis F | GGGAGAGGTTAGTTTATAGG |
| *CDKN1C* enhancer 2 Bis R (Bio) | ACTATCCCCAACCTATACTATCCAA |
| *CDKN1C* enhancer 2 Seq F | GTTTTTATTTTTGTTTTT |
| *CDKN1C* enhancer 1 Bis F | GGTAGGGTGGGTTTTGTAGTTAG |
| *CDKN1C* enhancer 1 Bis R (Bio) | ACCCCTAACAACAAAAACTAA |
| *CDKN1C* enhancer 1 Seq F | GGGTGTTGGAAGAGTAAG |
| KCNQ1OT1:TSS-DMR Bis F | TGTTTTTGTAGTTTATATGGAAGGG |
| KCNQ1OT1:TSS-DMR Bis R (Bio) | AACATACCAAACCACCCACCTAACAAAA |
| KCNQ1OT1:TSS-DMR Seq F | ATGGTAATGTTTGGTATTTAGAA |
| **ChIP PCRs** |  |
| H19/IGF2:IG-DMR ChIP F | TCCTTCGGTCTCACCGCCTG |
| H19/IGF2:IG-DMR ChIP R | TTCCACGGGCGAACCCCAGT |
| KCNQ1OT1:TSS-DMR ChIP F | ACTGGCTGGGTGTGAGGTGGCGCGA |
| KCNQ1OT1:TSS-DMR ChIP R | CACGCAGAGAACTGGCCCGTGTC |
| CDKN1C promoter ChIP F | TCCTCGAGGCCGTCGAGGGACT |
| CDKN1C promoter ChIP R | GATTACGACTTCCAGCAGGACAT |
| CDKN1C enhancer 3 ChIP F | GGACGGCTGTTGGGTTCTTG |
| CDKN1C enhancer 3 ChIP R | AGCACTCCTTGGCGGGTGCCA |
| CDKN1C enhancer 2 ChIP F | AGACCCGGGAGAGGTCAGCTCA |
| CDKN1C enhancer 2 ChIP R | CCATGCCAACAACACAGGGTAC |
| CDKN1C enhancer 1 ChIP F | CTTCCAGGGAAATGGGCGTTTCC |
| CDKN1C enhancer 1 ChIP R | AGCGTCCAGGGTGTGATTCCAGGCA |
| **3C PCRs** |  |
| HindIII 3C CDKN1C F | CACTGAGTTTCAGCAGAGATTAAAC |
| HindIII 3C CDKN1C R | GCTGCCGCAGAGCAGCGTTCGG |
| HindIII 3C 3 enhancer F | TGGCCCACAGGGTGGCTCCCT |
| HindIII 3C 3 enhancer R | GGCTGCATGGTCTCCATGGCAGG |
| HindIII 3C 2 enhancer F | CAGGCCCCTGCCGGTGAGTAGAC |
| HindIII 3C 2 enhancer R | AGTGACAGTGTCTGCCCTCGGGA |
| HindIII 3C 1 enhancer F | CAGCTGCTGCTGAGACAGTGCA |
| HindIII 3C 1 enhancer R | ACAGAAGCACTGGATTGTGGACA |
| HindIII 3C KCNQ1 promoter F | AGCTGAGAGACAGCAGATGCCCA |
| HindIII 3C KCNQ1 promoter R | GGTCTGGGTCAAAGTGGCCCAGG |
| HindIII 3C 3’KCNQ1OT1 F | AGTATCATATCTCCGTTAAACA |
| HindIII 3C 3’KCNQ1OT1 R | GGCAGATTTCATACTCTTCTG |
| HindIII 3C 3’KCNQ1OT1:TSS-DMR F | ACCAGACCCTGGGTGGGGGCCGA |
| HindIII 3C 3’KCNQ1OT1:TSS-DMR R | GGCCTTTCTCCTGCTAATTGTAG |
| HindIII 3C intragenic CpGi F | GGAGGTGGGAATTAATGAAAG |
| HindIII 3C intragenic CpGi R | TCGTCTGTGGCGGGCACTGGGGCT |
| HindIII 3C SLC22A18 promoter F | TCCTTGCAGGTGCCCGCAGCTTCT |
| HindIII 3C SLC22A18 promoter R | CACTAAACTAACATGAATTTGCC |
