# Supplementary material for: Epigenetic Characterization of CDKN1C in Placenta Samples from Non-syndromic Intrauterine Growth Restriction
Source: Front Genet. 2016 Apr 26;7:62. doi: 10.3389/fgene.2016.00062 (PMC4844605; doi:10.3389/fgene.2016.00062)
Supplement: Table S2 — Summary of the experimental results that based our selection for internal control genes in placental tissue. MRPL19, mitochondrial ribosomal transcript; GUS3, glucuronidase 3; HPRT, hypoxanthine guanine phosphoribosyl transferase; NCL, nucleolin; GAPDH, glyceraldehyde-3-phosphate dehydrogenase; MRLPL19 showed the smallest variation between placental samples and the closest amplification characteristics (cycle threshold, CT) to those of our genes of interest. [file Table_2.DOCX]

|  | **Average C_T_ (AvgC_T_)** | **Range of AvgC_T_ in a set of 4 placentae** |
| --- | --- | --- |
| β-actin | 18 | 18.3-20.9 |
| **L19** | **21** | **21.3-22.3** |
| GUS3 | 28 | 27.4-29.1 |
| HPRT | 25 | 25.7-27.4 |
| NCL | 26 | 23.4-29.2 |
| GAPDH | 24 | 23.7-25.7 |
