# Supplementary material for: Epigenetic Characterization of CDKN1C in Placenta Samples from Non-syndromic Intrauterine Growth Restriction
Source: Front Genet. 2016 Apr 26;7:62. doi: 10.3389/fgene.2016.00062 (PMC4844605; doi:10.3389/fgene.2016.00062)
Supplement: Table S3 — A summary of non-significant variables that are not associated with changes in CDKN1C expression levels. [file Table_3.DOCX]

| **Variable** | **P values (Student’s t-test)** |
| --- | --- |
| Gender | 0.993 |
| Pre-eclampsia | 0.111 |
| Presence of labor | 0.369 |
| Multiple pregnancy | 0.098 |
|  | **P value (ANOVA)** |
| Smoking (non-smoker/before but not during pregnancy/during pregnancy) | 0.430 |
|  | **P value (Pearson’s correlation)** |
| Maternal age | 0.592 |
| Maternal height | 0.797 |
| Pre-gestational weight | 0.506 |
| Gestational weight | 0.223 |
| Gestational age | 0.766 |
| Placenta weight | 0.134 |
| Birth weight  BW SDS | 0.199  0.095 |
| Birth length  BL SDS | 0.778  0.179 |
| Head circumference  HC SDS | 0.989  0.900 |
